# Amelioration of Experimental Autoimmune Encephalomyelitis in Alzheimer’s Disease Mouse Models: A Potential Role for Aβ

**DOI:** 10.3390/cells11061004

**Published:** 2022-03-16

**Authors:** Changjie Shi, Jiaxue Cha, Junyuan Gong, Shaodeng Wang, Peng Zeng, Junjiang Lian, Bowen Zhang, Qiuhong Hua, Jie Lv, Changsheng Du, Xin Xie, Ru Zhang

**Affiliations:** 1 Shanghai Key Laboratory of Signaling and Disease Research, Laboratory of Receptor-Based Bio-Medicine, Collaborative Innovation Center for Brain Science, School of Life Sciences and Technology, Tongji University, Shanghai 200092, China; 11006@tongji.edu.cn (C.S.); chajiaxue@tongji.edu.cn (J.C.); 1831520@tongji.edu.cn (J.G.); shaodengwang@tongji.edu.cn (S.W.); 1410778@tongji.edu.cn (P.Z.); 1710547@tongji.edu.cn (J.L.); 1810545@tongji.edu.cn (B.Z.); 96811@tongji.edu.cn (Q.H.); 18310109@tongji.edu.cn (J.L.); duchangsheng@tongji.edu.cn (C.D.); 2 CAS Key Laboratory of Receptor Research, The National Center for Drug Screening, Shanghai Institute of Materia Medica, Chinese Academy of Sciences, Shanghai 201203, China; xxie@simm.ac.cn

**Keywords:** amyloid-β peptide, experimental autoimmune encephalomyelitis, multiple sclerosis, Alzheimer’s disease, Th17

## Abstract

Emerging data have highlighted the coexistence of multiple sclerosis (MS) and Alzheimer’s disease (AD), both of which are common central nervous system degenerative diseases with a heavy burden on patients, their families, and society. However, it is unclear how MS progresses under an AD pathological background. We aimed to address the question of how MS progresses under an AD pathological background. We induced the experimental autoimmune encephalomyelitis (EAE) model of MS in two types of AD mouse models, Tg6799 and APP/PS1 mice. We found that, compared with wild-type mice, the clinical symptoms of EAE were significantly ameliorated in APP/PS1 mice but not in Tg6799 mice. Moreover, a much lower level of serum Aβ was observed in Tg6799 mice. EAE clinical symptoms in Tg6799 and C57BL/6J mice were ameliorated by intraperitoneal injection of Aβ42. Peripheral administration of Aβ42 peptides was able to inhibit Th17 development in vivo, which is likely to occur through the inhibition of IL-6 production in dendritic cells. Our findings revealed that AD and EAE could coexist in the same mouse, and Aβ residing in peripheral circulation likely plays an anti-inflammatory role in preventing EAE progression. These findings reveal the potential benefit of Aβ, one of the supervillains of AD, at least in certain contexts.

## 1. Introduction

Multiple sclerosis (MS) is a chronic neuroinflammatory and demyelinating autoimmune disorder of the central nervous system characterized by damage to neurons and their protective myelin sheath in both the brain and spinal cord. This disorder often causes disability in young people. MS is a predominantly T-cell mediated autoimmune disorder, and CD4+ T cells, especially the Th17 and Th1 subgroups, have been suggested to cause the early initiation of the disease [1]. Experimental autoimmune encephalomyelitis (EAE) is one of the widely used animal models of MS [2,3], which shares many clinical, pathological, and histological similarities with MS, such as inflammation and axonal degeneration [4].

Alzheimer’s disease (AD) is the most common form of dementia, with extracellular amyloid plaque deposition and neuron damage as the primary pathological hallmarks. It is widely accepted that amyloid β (Aβ) accumulation in hippocampal and cortical regions of the brain is critical to neurodegeneration and inflammation in AD [5,6,7]. Aβ peptides, mainly Aβ40 and Aβ42, are derived from the sequential processing of the transmembrane amyloid precursor protein (APP) by β- and γ- secretase [8,9,10]. Aβ accumulation is generally considered detrimental to human health; however, a few reports have indicated various physiological functions of Aβ [11]. Interestingly, previous reports showed that Aβ expression could protect against microbial infection in AD mice [12], and peripheral administration of Aβ led to a reversal of paralysis and reduced inflammation in a multiple sclerosis mouse model [13]. The latter raises the question of whether MS and AD can coexist.

MS and AD are both relatively common CNS degenerative diseases that result in a heavy burden on patients, their families, and society. However, there is limited information concerning the coexistence of MS with AD. Recently, one study analyzed PubMed citations and found that MS is frequently co-cited with Alzheimer’s, indicating that MS is closely associated with Alzheimer’s disease [14]. A recent review tried to explore the coexistence of MS and AD via a focused literature search to identify any reports of individuals with both MS and AD [15]. Twenty-four individuals were found with pathological features of both MS and AD in case series or reports, which provide evidence that AD and MS can coexist in the same person. However, little is known concerning how AD influences MS, or vice versa.

In this study, we aimed to address the question of how MS progresses under an AD pathological background. EAE was established in two types of AD mouse models, APPswe/PSEN1ΔE9 (APP/PS1) and 5xFAD transgenic (Tg6799) mice, and we found that the clinical symptoms of EAE were significantly ameliorated in APP/PS1 mice compared to their wild-type (WT) littermates. Unexpectedly, the alleviation of EAE was not observed in the other AD mouse model, Tg6799 transgenic mice. Interestingly, further investigations revealed that the serum Aβ level in Tg6799 mice was relatively low, and EAE clinical symptoms of Tg6799 mice were ameliorated by intraperitoneal injection of Aβ42. Similar results were obtained in C57BL/6J mice after peripheral administration of Aβ42 peptides. Our observations indicated that under an AD pathological background, the serum soluble Aβ level rather than insoluble senile plaques of Aβ in the brain had an important role in ameliorating EAE. Furthermore, we found that exogenous administration of Aβ42 peptides could inhibit Th17 development and IL-6 production by DCs, which might therefore block EAE inflammation in vivo.

## 2. Materials and Methods

### 2.1. Animals and Reagents

APP/PS1 transgenic mice (Jackson Laboratory, Bar Harbor, ME, USA, stock number 004462) express a chimeric mouse/human amyloid precursor protein (Mo/HuAPP695swe) and a mutant human presenilin 1 (PS1-dE9). 5xFAD transgenic mice (Tg6799, Jackson Laboratory) overexpress mutant human amyloid-beta precursor protein 695 (APP) with the Swedish (K670N, M671L), Florida (I716V), and London (V717I) familial Alzheimer’s disease (FAD) mutations, along with human presenilin 1 (PS1) harboring two FAD mutations, M146L, and L286V.

C57BL/6J mice (Shanghai Laboratory Animal Center, Shanghai, China), MOG35-55 (MEVGWYRSPFSRVVHLYRNGK, GL Biochem, Shanghai, China), and Aβ42 (purity > 95%, GL Biochem, Shanghai, China) were purchased as indicated. Human β amyloid 40/42/total ELISA kits were obtained from Shanghai ExCell Biology, Inc., Shanghai, China. Thioflavin S was obtained from Sigma, St. Louis, MO, USA. All experimental procedures were approved by the Tongji University Animal Care Committee.

### 2.2. EAE Induction and Intraperitoneal Injection of Aβ42

EAE was induced in 4-month-old female Tg6799, APP/PS1, and C57BL/6J mice by administering 200 μg of MOG35-55 in complete Freund’s adjuvant that contained 5 mg/mL heat-killed Mycobacterium tuberculosis H37RA. Pertussis toxin (200 ng/mouse) was injected intraperitoneally on day 0 and day 2. EAE severity was evaluated as described in [16,17,18]. Briefly, EAE clinical signs were scored every day as follows: 0, normal; 1, only paralyzed tail; 2, paresis (weakness or partial paralysis of hind limbs); 3, paraplegia (totally paralyzed hind legs); 4, paraplegia with forelimb weakness or paralysis; and 5, moribund or death. For Aβ42 experimental interventions, 10-week-old C57BL/6J and Tg6799 mice received injections of 50 μg Aβ42 peptides [13] or PBS twice a day from day 5 or day 12 until the experimental endpoint. To evaluate the therapeutic effects of Aβ42, Aβ42 treatment was started on day 12 after MOG immunization, when EAE mice developed mild clinical symptoms. Next, to further explore the preventive and therapeutic effects of Aβ42, Aβ42 treatment was started on day 5 after MOG immunization, prior to the onset of clinical symptoms.

### 2.3. Histopathological Analysis and Thioflavin-S Staining of Senile Plaques

For histological staining, mice were anesthetized and perfused with PBS at the end of the experiment. The spinal cords and left hemibrains were carefully removed and fixed in 4% (*w*/*v*) paraformaldehyde overnight. The samples were then soaked in 30% sucrose (in PBS) for 24 h and embedded in optimum cutting temperature compound (OCT). Then, 14 μm spinal cord sections were collected for staining with hematoxylin and eosin (H&E) to analyze infiltrating cells. Thioflavin-S staining was performed on 30 μm brain sections of 4-month-old mice. Briefly, the brain sections were rinsed in PBS three times for 5 min each, immersed in 0.1% (*w*/*v*) thioflavin S for 8 min, and then washed in PBS and 70% alcohol for 5 min each.

### 2.4. ELISA Analysis

First, we isolated the cerebral cortex and hippocampus from the right hemibrains. Subsequently, Aβ40 and Aβ42 in the SDS and FA extracts of the brain tissues were measured by ELISA. Orbital blood was collected from the mice in each group, and serum was isolated. The serum Aβ levels were determined by using human total Aβ ELISA kits. The serum IL-6 levels were quantified by ELISA following the manufacturer’s instructions.

### 2.5. CD4+ T Cell Separation and In Vitro Differentiation

Naive CD4+ T cells were isolated from spleens of 8-week-old female C57BL/6J mice as previously described [19]. Cells were activated with anti-CD3 (2 μg/mL) and anti-CD28 (2 μg/mL). For Th1 differentiation, cells were stimulated with IL-12 (10 ng/mL) and anti-IL-4 (10 μg/mL). For Th17 differentiation, cells were treated with anti-IL-4 (10 μg/mL) and anti-IFN-γ (10 μg/mL), together with a cytokine mixture containing IL-6 (30 ng/mL), IL-23 (10 ng/mL), IL-1β (10 ng/mL), TNF-α (10 ng/mL), and TGF-β1 (3 ng/mL). Treg polarization was induced by adding anti-IFN-γ (10 μg/mL), IL-2 (10 ng/mL), and TGF-β1 (5 ng/mL).

### 2.6. DC Isolation, Stimulation, IL-6, and Cell Toxicity Measurement

Mouse CD11c+ DC cells were isolated from the lymph nodes of mice by MACS (Miltenyi Biotec, Bergisch Gladbach, Germany). Then, DCs were stimulated with anti-CD40 (10 μg/mL). IL-6 was measured in the supernatants of cultured DCs receiving no treatment or receiving treatment with anti-CD40 alone or in combination with Aβ42 (0.5, 5, or 50 ng/mL). Cell Counting Kit-8 was used to test cell toxicity.

### 2.7. Flow Cytometry

The percentage of CD4 + Foxp3+ Tregs was analyzed using flow cytometry (FACS). The percentage of Th1 or Th17 cells was evaluated by FACS; only cells positive for IFN-γ (IL-17-) were defined as Th1 cells, whereas cells positive for IL-17 (IFN-γ-) were considered Th17 cells.

### 2.8. Statistical Analysis

Data are expressed as mean ± SEM. A two-way ANOVA test was applied to analyze the significance of EAE clinical scores. Student’s *t*-test was used to assess other analyses, and *p* values less than 0.05 (*p* < 0.05) were considered statistically significant.

## 3. Results

### 3.1. Clinical Symptoms Were Ameliorated When EAE Was Induced in APP/PS1 Mice, but Not in Tg6799 Transgenic Mice

**Figure 1:**
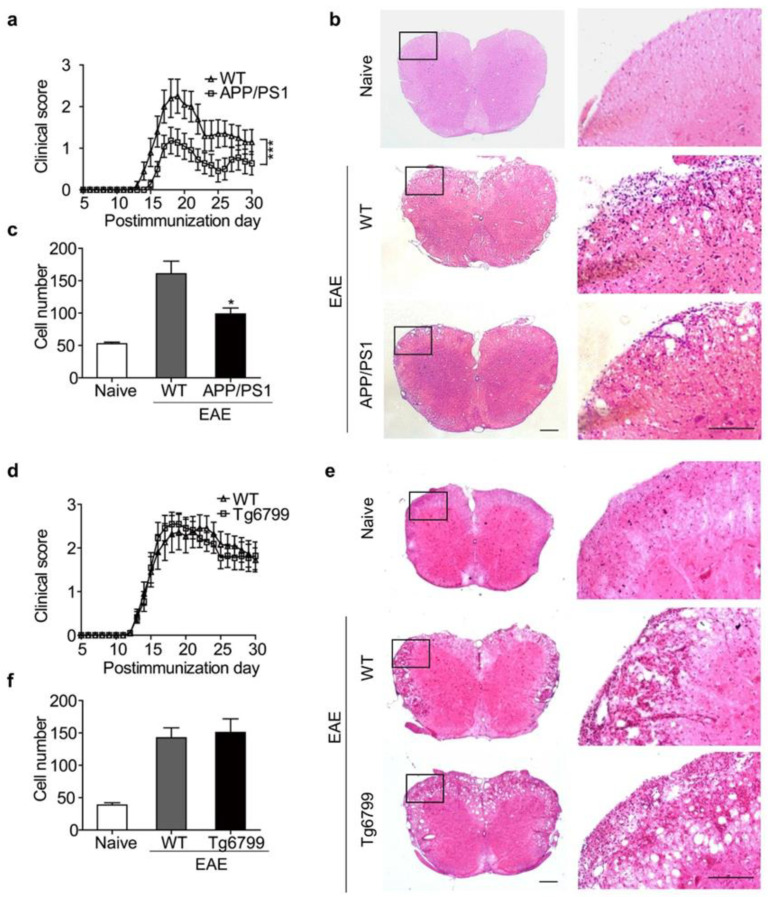
Clinical symptoms of EAE in APP/PS1 and Tg6799 transgenic mice. (**a**) Clinical scores of EAE in WT and APP/PS1 mice. Results are presented as daily mean clinical scores ± SEM. *** *p* < 0.001, two-way ANOVA (WT, *n* = 10; APP/PS1, *n* = 11). (**b**) Hematoxylin and eosin (H&E)-stained slides of spinal cords from naive, WT, and APP/PS1. (**c**) Quantification of infiltrating cells. Data are mean ± SEM, * *p* < 0.05 (Student’s *t*-test). (**d**) Clinical scores of EAE in WT and Tg6799 mice (*n* = 11 per group). (**e**,**f**) H&E staining was performed to analyze infiltrating cells. The boxed areas in the left columns are presented enlarged on the right. Scale bars, 200 μm.

To investigate how MS progresses in AD, we examined the progression of EAE in two popular AD models, Tg6799 and APPswe/PS1Δ9 (APP/PS1) mice. We induced EAE with MOG35–55 in 4-month-old AD mice and their littermates (wild type, WT) at the same age. WT mice exhibited a typical course of EAE and began to develop clinical signs on day 14 postimmunization. Compared with WT, the onset of EAE in APP/PS1 mice was delayed until day 16 postimmunization, and the peak severity and cumulative clinical score were significantly reduced in APP/PS1 mice (Figure 1a). Surprisingly, this amelioration of EAE clinical symptoms was not observed in Tg6799 AD mice (Figure 1d).

Histological examination of the spinal cord was performed on day 30 postimmunization. The WT mice showed typical leukocyte infiltration in the spinal cord, whereas the APP/PS1 mice had much fewer infiltrating leukocytes in the spinal cord (Figure 1b,c). Consistent with the previous clinical score, Tg6799 mice showed similar cell infiltration to WT mice (Figure 1e,f). These results indicate that some beneficial factors preventing EAE exist in APP/PS1 mice but not in Tg6799 AD mice.

### 3.2. Distinct Aβ Pathology in APP/PS1 and Tg6799 Mice

**Figure 2:**
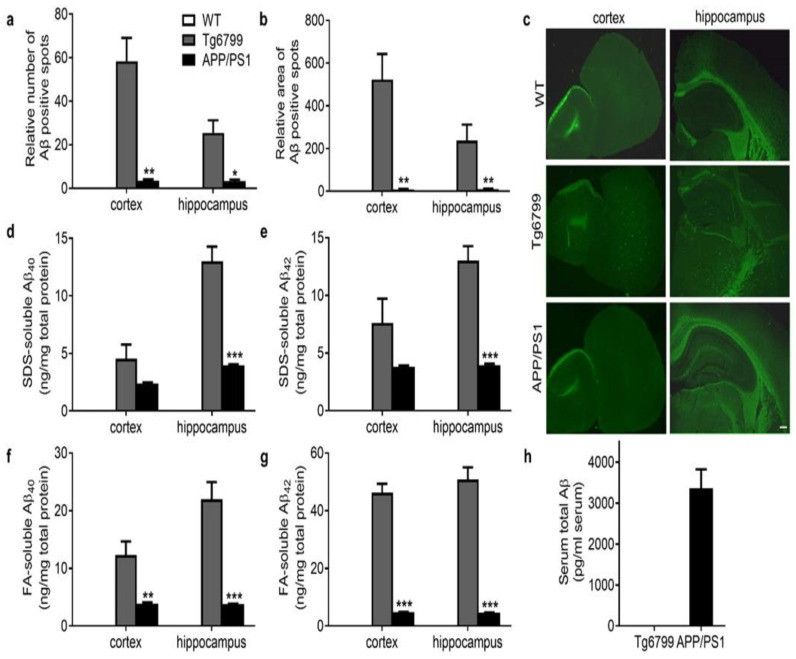
Serum and brain Aβ levels in Tg6799 and APP/PS1 transgenic mice. (**a**,**b**) The relative number and area of Aβ plaques in cortex and hippocampus of WT, Tg6799, and APP/PS1 mice were quantified. (**c**) Representative images of senile plaques with thioflavin-S staining in cortex and hippocampus sections. (**d**–**g**) Brain Aβ levels, including (**d**,**e**) SDS-soluble Aβ42 and Aβ40 and (**f**,**g**) FA-soluble Aβ42 and Aβ40 in the cortex and hippocampus of WT, Tg6799, and APP/PS1 mice by ELISA. *n* = 3–5. Data are mean ± SEM, *** *p* < 0.001, ** *p* < 0.01, * *p* < 0.05 (Student’s *t*-test). (**h**) Serum Aβ levels in Tg6799 and APP/PS1 transgenic mice were measured by using the human total Aβ ELISA kits. *n* = 6. Scale bars, 200 μm.

APP/PS1 and Tg6799 are both commonly used AD mouse models showing the age-related progression of Aβ plaque deposits in the brain and cognitive decline [20]. To gain insight into the factors responsible for their different reactions to EAE, we first performed a detailed investigation of Aβ pathology in these mice. Thioflavin-S staining revealed large Aβ plaque deposits in the cortex and hippocampus in 4-month-old Tg6799 mice (Figure 2a–c) but not in WT mice. In APP/PS1 mice, only a few Aβ plaque deposits in the brain were visible at this age. When using SDS and FA to lyse the brain, Aβ40/42 accumulation was observed in both Tg6799 and APP/PS1 mice, but much higher Aβ levels were detected in Tg6799 (Figure 2d–g). This phenomenon is consistent with a previous report showing accelerated plaque development and even earlier intraneuronal Aβ42 accumulation at 1.5 months of age in Tg6799 strains [21]. Furthermore, we measured the serum Aβ level in Tg6799 and APP/PS1 mice using ELISA (Figure 2h). Unexpectedly, the serum Aβ level was nearly undetectable in Tg6799 mice, whereas in APP/PS1 mice of the same age, the serum total Aβ reached 3355 pg/mL on average. This result raises the possibility that the serum Aβ, rather than that in the brain, leads to a reduction in paralysis of EAE in APP/PS1 mice. This suggestion is reminiscent of a previous report showing that peripheral administration of Aβ in mice could alleviate EAE [13].

### 3.3. Intraperitoneal Administration of Aβ42 Peptides Ameliorated the Clinical Symptoms of EAE in Tg6799 Mice

**Figure 3:**
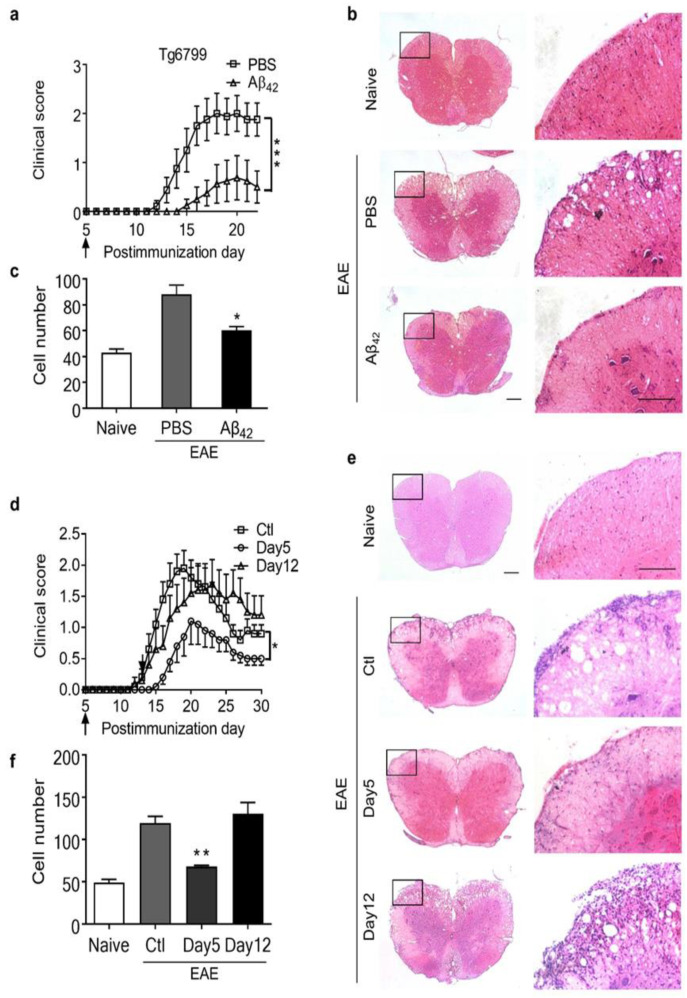
Reduction in EAE clinical symptoms by intraperitoneal injection of Aβ42 peptides. (**a**) Clinical scores of EAE mice treated with Aβ42 peptides or PBS in Tg6799 mice. EAE was induced in 10-week Tg6799 mice by immunization with MOG35–55 peptide, Aβ42 peptides, or PBS, which were administered daily via intraperitoneal injection from day 5 postimmunization. Data are mean ± SEM, *** *p* < 0.001(PBS group, *n* = 9; Aβ42 group, *n* = 9). (**b**,**c**) H&E staining of spinal cord sections and quantification of infiltrating cells. * *p* < 0.05 (Student’s *t*-test). (**d**) Clinical scores (mean ± SEM) of MOG-immunized mice treated with Aβ42 peptides or PBS in 10-week C57BL/6J mice. Aβ42 peptides or PBS were administered daily via intraperitoneal injection from day 5 or day 12 postimmunization. * *p* < 0.05 (*n* = 10). (**e**,**f**) H&E staining of spinal cord sections and quantification of infiltrating cells. Data are mean ± SEM, ** *p* < 0.01 (Student’s *t*-test). The boxed areas in the left columns are presented enlarged on the right. Scale bars, 200 μm.

To test whether the serum Aβ level is one of the key factors interfering with EAE severity, systemic administration of Aβ was carried out before EAE onset in Tg6799 mice. Either 50 μg of synthesized Aβ42 peptides or the PBS vehicle control was administered twice daily via intraperitoneal injection from day 5 post-EAE induction until the end of the study. Strikingly, Aβ42 peptide administration was able to not only significantly reduce the peak severity and cumulative clinical score of EAE but also delay the onset of the disease in Tg6799 mice (Figure 3a–c).

To determine whether the protective effect of Aβ treatment on EAE was restricted to the AD models, we treated C57BL/6J mice with Aβ42 or PBS twice daily from day 5 or day 12 postimmunization. We found that peripheral injection of Aβ42 from day 5 postimmunization could eliminate EAE clinical symptoms, as well as infiltrating cells, in normal mice (Figure 3d–f). More interestingly, the protective effect of Aβ against EAE was dependent on the time point of administration. When administered after the onset of the disease (day 12 postimmunization), Aβ42 was unable to reduce the severity of EAE in C57BL/6J mice (Figure 3d), which indicates a preventative effect rather than a therapeutic benefit of this peptide in these mice.

### 3.4. Aβ42 Regulated Th17 Development by Reducing IL-6 Production by DCs

**Figure 4:**
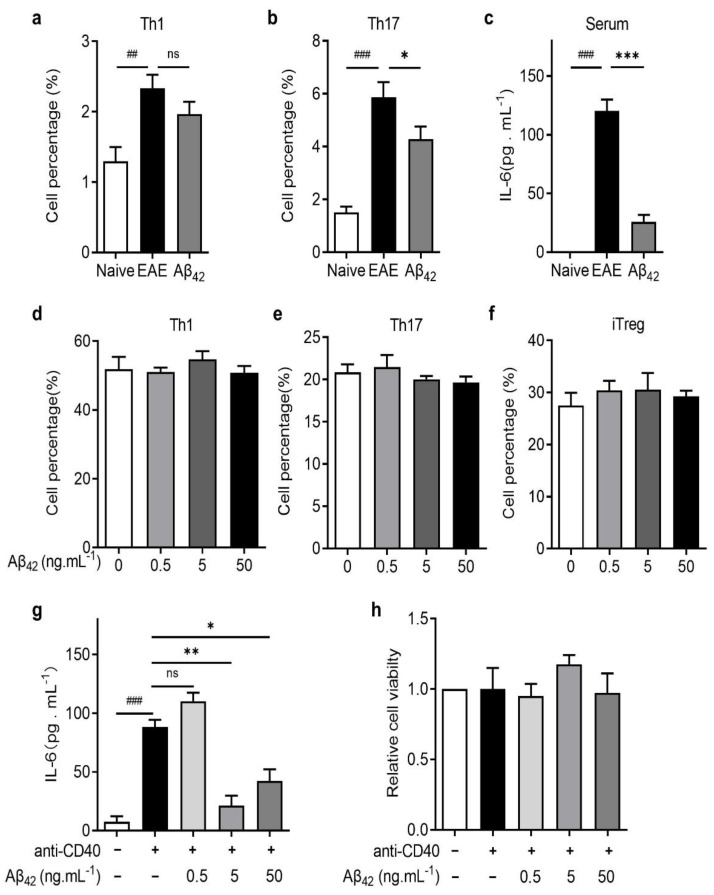
Aβ42 reduced IL-6 production and inhibited Th17 development in vivo. (**a**,**b**) Th1 and Th17 cells were analyzed in vivo. (**c**) The levels of IL-6 in serum of naïve mice, EAE mice, and Aβ-treated EAE mice were measured in vivo. (**d**–**f**) Naive CD4+ T cells isolated from C57BL/6J mice were induced to differentiate into Th1, Th17, and iTreg cells with the addition of different concentrations of Aβ42 (0.5, 5, or 50 ng/mL) in vitro. (**g**,**h**) The IL-6 level and the relative viability of dendritic cells were measured by ELISA in vitro. Data are presented as mean ± SEM, *** *p* < 0.001, ** *p* < 0.01, * *p* < 0.05, ^###^ *p* <  0.001, ^##^ *p* <  0.01, ns: not significant (*n* = 3–5, Student’s *t*-test).

Th1 and Th17 cells are known to be the main pathogenic CD4+ T effector cells in EAE [22]. To gain further insight into the mechanism of Aβ42 protective effects in EAE, we analyzed the subpopulation of CD4+ T cells in the spleen of Aβ42-treated or untreated mice. Compared to PBS-treated EAE mice, the percentage of Th17 cells in the CD4+ cells was significantly lower in Aβ42-treated mice, whereas no significant change in the Th1 percentage was observed (Figure 4a,b). Next, we tested whether Aβ42 directly affects T cell differentiation using an in vitro T cell differentiation assay. Naïve CD4+ cells were isolated from the spleen of C57BL/6J mice and induced to differentiate into Th1, Th17, or Tregs under the corresponding polarization conditions. As shown in Figure 4d–f, administration of Aβ42 did not affect Th1, Th17, or iTreg differentiation in vitro.

IL-6 is a critical pro-inflammatory cytokine for Th17 development [23]. Indeed, the serum concentration of IL-6 was significantly increased in PBS-treated EAE mice but decreased dramatically after Aβ42 treatment (Figure 4c). DCs are the main antigen-presenting cells (APCs) in the periphery, which produce potent pro-inflammatory molecules upon maturation [24]. We then tested whether Aβ42 can affect IL-6 production by DC cells. In the in vitro IL-6 production assay, DCs isolated from the lymph nodes of naive mice were stimulated with anti-CD40 alone or in combination with Aβ42 at various concentrations. The anti-CD40 treatment enhanced IL-6 production by DCs, and Aβ42 at 5 ng/mL and 50 ng/mL significantly decreased the anti-CD40-stimulated IL-6 production to nearly 75% and 50% (Figure 4g), without affecting the relative viability of DCs (Figure 4h). These data suggest that Aβ42-mediated reduction in the Th17 subpopulation in vivo does not arise from a direct effect on T cell differentiation but is an indirect effect via modulation of upstream cytokine production by APCs.

## 4. Discussion

Alzheimer’s disease and multiple sclerosis are relatively common neural degenerative diseases that cause CNS neuronal damage and activate inflammation. Due to the extended potential life span of humans and advancements in therapeutic strategies, people with MS are living longer than ever and will likely face the same age-related diseases, such as AD, as other seniors. In this study, we investigated EAE progression in two transgenic AD models, both with abundant Aβ plaque deposits in the brain. Interestingly, by inducing EAE in two AD mouse models, we found that MS clinical symptoms could still be established under an AD pathological background, which agrees with previous case reports of the coexistence of AD and MS in the same person [14,15].

More surprisingly, the clinical severity of induced EAE was distinct in the two AD models, which correlated with the serum Aβ42 level in these mice but not with brain Aβ plaques. Both AD mice with a high serum level of Aβ42 (APP/PS1) and mice with undetectable serum Aβ42 (Tg6799) but receiving Aβ42 peripherally showed alleviated EAE symptoms. These results not only showed us how MS-like disease might progress under an AD background but also reflected the two sides of Aβ. Aβ is mostly considered one of the supervillains of AD; however, it is highly conserved throughout evolution and may have crucial physiological roles [25]. The bad reputation of Aβ stems from its propensity to form aggregates called amyloid plaques in the brain that are regarded as characteristic pathological features of AD [26]. Current therapeutic efforts have been aimed at reducing the detrimental insoluble Aβ42 peptide, but little attention has been devoted to its soluble precursor, which is proposed to have various physiological functions, such as regulating learning and memory, angiogenesis, and neurogenesis; repairing leaks in the blood–brain barrier; promoting recovery from injury; acting as an antimicrobial peptide and tumor suppressor; and modulating mitochondrial function and even neuroprotection [25]. Recent publications have shown that amyloid-positive individuals with normal cognition have higher soluble Aβ42 levels in the brain than those with mild cognitive impairment or dementia, and higher soluble Aβ42 levels in the human brain were associated with better cognitive performance and greater hippocampal volume [27,28]. Consistent with these reports, our findings in the current study showed that under an AD pathological background, the serum soluble Aβ level rather than insoluble senile plaques of Aβ in the brain had an important role in ameliorating EAE, supporting that the soluble, non-aggregating forms of Aβ42 have some positive effects.

It was reported that blood Aβ levels were increased before dementia onset in patients with familial AD [29], and in some patients, the blood Aβ42 level presented an initial increase followed by a decrease before and during the early stages of AD [27]. However, these patterns of blood Aβ in AD patients have not been found in other clinical analyses [30,31,32]. Nevertheless, many studies have been working on using the variation in Aβ42 in the blood as an early diagnostic biomarker of AD prior to the appearance of clinical symptoms [33,34]. Consistent with our study, others reported that the serum Aβ42 level reached 900 pg/mL in 3-month-old APP/PS1 mice prior to the appearance of learning and memory deficits [35]. At present, the factor that leads to the especially high level of serum Aβ in APP/PS1 mice is unclear. Amyloid-β peptide is a substrate of the human 20S proteasome, and the decline in proteasome function associated with aging would contribute to increased Aβ levels [36,37,38]. Substantial evidence also points to ubiquitin–proteasome system (UPS) malfunction as an important factor playing a key role in Aβ amyloid growth and AD pathogenesis [39,40]. Therefore, proteasomal dysfunction could be one of the additional factors promoting Aβ accumulation, which needs to be further investigated in the future.

In the current study, we found that serum Aβ in AD mice conferred protection against EAE by regulating the balance between Th1, Th17, and iTreg, which are CD4+ T effector cells that have a pivotal role in the pathogenesis of MS and EAE [41]. We found that the administration of Aβ42 can impair Th17 cell development, probably through a reduction in IL-6, an important cytokine secreted from DCs that governs the differentiation of Th17 cells in the development of EAE [42,43,44]. Therefore, at least in EAE, serum Aβ plays a beneficial role against the disease. A previous report also showed that peripheral administration of Aβ could reverse paralysis and reduce inflammation in an EAE model [13]. Others also reviewed the literature and reported that Aβ might regulate learning and memory, angiogenesis, and neurogenesis; repair leaks in the blood–brain barrier; promote recovery from injury; and act as an antimicrobial peptide and tumor suppressor in certain contexts [25]. How the serum Aβ level in AD correlates with AD diagnosis and prognosis remains elusive and is worthy of further investigation. Comprehensive consideration of both the physiological and pathological roles of Aβ under certain circumstances might be essential for the design of safe and effective therapeutic strategies for certain diseases.

## 5. Conclusions

We revealed that AD and EAE could coexist in the same mouse, and the clinical symptoms of EAE were ameliorated in APP/PS1 AD mice but not in 5xFAD transgenic (Tg6799) AD mice. The Aβ residing in the peripheral circulation plays an anti-inflammatory role in preventing EAE progression in AD mice. We highlight a potentially beneficial role for Aβ, and the findings may be of benefit to clinical diagnosis or therapy of both neurodegenerative diseases.

## Data Availability

All data generated or analyzed during this study are included in this published article and are available from the corresponding author upon reasonable request.
